# Editorial: Fruit Ripening: From Present Knowledge to Future Development

**DOI:** 10.3389/fpls.2019.00545

**Published:** 2019-05-01

**Authors:** José M. Palma, Francisco J. Corpas, Luciano Freschi, Victoriano Valpuesta

**Affiliations:** ^1^Group of Antioxidants, Free Radicals and Nitric Oxide in Biotechnology, Food and Agriculture, Estación Experimental del Zaidín, CSIC, Granada, Spain; ^2^Departamento de Botânica, Instituto de Biociências, Universidade de São Paulo, São Paulo, Brazil; ^3^Departamento de Biología Molecular y Bioquímica, Instituto de Hortofruticultura Subtropical y Mediterránea (IHSM), Consejo Superior de Investigaciones Científicas- Universidad de Málaga, Málaga, Spain

**Keywords:** fruit ripening, transcriptomics, metabolomics, fruit quality, hormones

Fruit ripening is a very well-orchestrated physiological process of Angiosperm species which is under developmental, hormonal and epigenetic regulation and is finely tuned by environmental stimuli (Figure 1; Palma et al., 2011; Bianchetti et al., 2018; Corpas et al., 2018). Over the years, a number of signaling molecules (e.g., phytohormones) have been implicated in controlling fruit ripening, some of them playing very well-recognized roles like ethylene, abscisic acid (ABA) and reactive oxygen species (ROS), and others emerging only more recently as driving forces of this physiological process, such as nitric oxide (NO) and NO-derived molecules (reactive nitrogen species, RNS), hydrogen sulfide (H_2_S) and melatonin (Corpas and Palma, 2018; Corpas et al., 2018, 2019; Mukherjee, 2019). Among these newcomers, NO has received comparatively more attention, and it has been found that during fruit ripening this species promotes post-translational modifications (PTMs) through protein nitration and protein *S*-nitrosation of proteins (Corpas and Palma, 2018). Globally, fruit ripening has been a main focus of the plant research community, motivated not only by its biological and evolutionary significance in seed development and dispersal, but also by its implications on determining the quality and nutritional value of some of the most worldwide consumed foods (Agius et al., 2005; Palma et al., 2015, 2018; Karasawa and Mohan, 2018).

**Figure 1:**
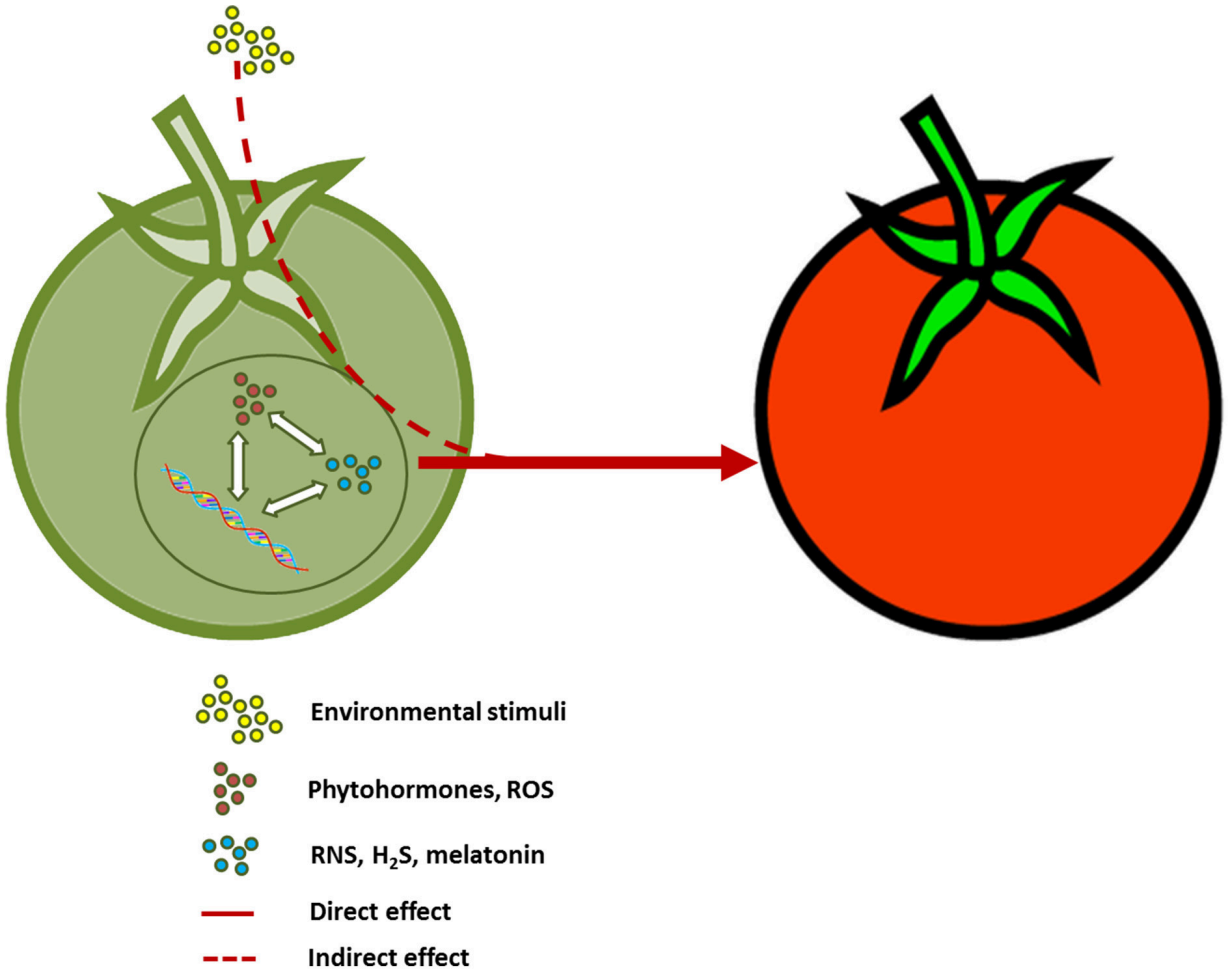
Integrated model for fruit ripening. A close interaction occurring in immature fruits among phytohormones, reactive oxygen and nitrogen species (ROS and RNS, respectively), hydrogen sulfide (H_2_S), melatonin and genes leads to a controlled physiological process (ripening) which can be also affected by the interaction with environmental stimuli.

From this overall view, many efforts have been dedicated by plant biologists to understanding fruit ripening, not only as a physiological phenomenon but also as a target to promote human health. This Research Topic is a faithful reflection of how plant research has been increasingly devoted to deciphering fruit ripening biology. Contributions made by 88 scientists from 11 countries belonging to 4 continents for this special issue have accumulated together more than 23,900 views until mid-March 2019. Using the most recent cutting-edge approaches, the authors have focused their work on major ripening-related topics, embracing distinct disciplines such as chemistry, physiology, genetics, biochemistry and molecular biology. Genome editing, metagenomics, metabolomic and transcriptomic profiles, transcriptional regulation, gene expression, histology, signaling processes, and the antioxidant metabolism have been some of the tools used to build up this compendium about fruit biology.

In line with the interest of expanding the investigation of the role exerted by hormones on fruit ripening, several papers included in this Research Topic have dealt with the ripening-associated signaling network. For example, the proteomic quantification of the main proteins involved in the ethylene signal transduction pathway has been achieved by Mata et al., revealing that transcriptomic and proteomic patterns of some, but not all, these proteins positively correlated during tomato fruit ripening. Moreover, by the exogenous addition of ABA to the non-climacteric bilberry (*Vaccinium myrtillus*) fruits, Karppinen et al. demonstrated that this phytohormone plays a significant role in regulating processes linked to ripening such as anthocyanin biosynthesis and cell wall modification. It was also proved that sugars, either sucrose or glucose, have minor regulatory functions in the ripening of bilberry fruits (Karppinen et al.), in contrast with other non-climacteric fruits such as strawberry and grape, where both these sugars promote fruit ripening in a hormone-like signaling manner in coordination with ABA (Jia et al., 2013, 2017). Plant hormones, particularly ethylene and auxin, and light signaling have been proposed to establish a dynamic crosstalk, being identified as essential regulators of carotenoid biosynthesis during tomato fruit ripening. In this Research Topic, the potential involvement of ethylene and auxins in light-mediated regulation of tomato fruit carotenogenesis was investigated by comparing the impacts of light treatments and the light-hyperresponsive *high pigment-2* (*hp2*) mutation on both carotenoid synthesis and hormonal signaling (Cruz et al.).

The quality as an index of the fruit physiological condition has also been covered by this Research Topic. The biosynthesis and regulation of the vitamin C content during fruit ripening has been reviewed, with the perspective of the critical role exerted by ascorbate in the activation of epigenetic mechanisms controlling cell differentiation and dysregulation events, which eventually might lead to the development of diverse types of cancer. Thus, the different strategies to boost the ascorbate contents in crops, with special emphasis on fruits, have being reviewed by Fenech et al. The profiles of distinct organic acids during fruit development and ripening are also analyzed as qualitative and quantitative traits of crop fruits. In this context, it appears that citrate and malate play major roles on those physiological processes, as they usually are accumulated in numerous climacteric and non-climacteric fruits (Batista-Silva et al.). The relationship between peach (*Prunus persica* L.) fruit quality and β-galactosidases (BGALs), which are cell wall hydrolases critically important for fruit softening, has also been analyzed. The down-regulation of two *BGAL* genes in peach via virus-induced gene silencing (VIGS) delayed fruit softening by reducing the activity of polygalacturonases and pectinmethylesterases, which are known to promote cell wall degradation (Liu et al.). The regulation of anthocyanin production-related gene expression was also addressed in this special issue. Transcriptomic analysis revealed a significant down-regulation of genes encoding phenylpropanoid and flavonoid biosynthetic enzymes in young fig (*Ficus carica*) fruits of the color mutant “Purple peel” compared to “Green Peel,” whereas a simultaneous up-regulation in almost all of the anthocyanin and flavonoid pathway-related genes took place in the mutant at the mature stage. Metabolomic data also showed that anthocyanins, particularly cyanidins, are the major responsible for the distinctive purple phenotype of the mutation (Wang et al.).

Another interesting perspective included in this special volume concerns the plant microbiome, which is a research theme that has been increasingly gaining attention in recent years. The plant microbiome is considered a key determinant for plant health and productivity, thus having profound impacts on fruit quality. The link between the host and the fruit-associated microbiome was investigated in watermelon (*Citrullus lanatus*) using the carbohydrate metabolism as an index of the beneficial consequences of such interaction. The use of the holobiont concept to incorporate the associated microbiomes to the breeding programs is proposed (Saminathan et al.). Finally, product quality was also the driving force behind the study on storage proteins in olive (*Olea europaea*). The accumulation of seed storage proteins of the 7S-type (β-conglutin), analyzed by biochemical and immunocytochemical methods, suggests that these molecular markers could be used to facilitate assessing the appropriate ripening stage of olives for commercial and industrial purposes (Zafra et al.).

Molecular aspects of fruit ripening regulation and technological advances to improve ripening-associated traits are also among the topics included in the present issue. For example, the regulation of gene expression and transcript abundance via non-coding small (microRNAs among them) and long chain RNA (lncRNA) was reviewed in the context of ovule, seed and fruit development and ripening (de Oliveira-Correa et al.). The regulation of a conserved kinase complex was demonstrated to participate in the control of the ripening process in tomato fruits. In tomato, the sucrose non-fermenting-1-related protein kinase 1 (SnRK1) was shown to interact with several transcription factors and this event regulated the expression of downstream ripening-related genes, thereby promoting fruit ripening (Yu et al.). Finally, in the context of applying genome editing tools to improve commercially relevant crop species, Martín-Pizarro and Posé discuss the use of CRISPR/Cas9 technology not only for functional research but also to generate plants with improved fruit quality traits (Martín-Pizarro and Posé).

All these subjects addressed in this Research Topic provide new and complementary information to the model depicted in Figure 1, but they also open up new windows for future investigation on fruit ripening, highlighting some of the most promising areas for both basic and applied research. Thus, the potential interactions among NO, H_2_S, H_2_O_2_ and melatonin depicts a scenario where many intermediate players can be envisaged including small regulatory molecules but also antioxidant and stress-related enzymes (Muñoz-Vargas et al., 2018; Corpas et al., 2019; Mukherjee, 2019). The precise modulation through these novel molecules in coordination with other endogenous signaling compounds (i.e., phytohormones) in the different ripening-associated events (e.g., change in firmness, degradation of chlorophyll, synthesis of new pigments and flavonoids, etc.), and how it is governed in either climacteric and non-climacteric fruits remain to be totally understood and some critical avenues for upcoming research on the regulation of fruit metabolism and development are presented in this issue. In the last years, the regulation of the ascorbate metabolism by NO, and H_2_S has initiated new ways to establish direct connection among these signaling molecules and the antioxidant biochemistry in plants (Rodríguez-Ruiz et al., 2017; Shan et al., 2018). Therefore, fitting molecules like ascorbate and other players implicated in redox homeostasis and signaling, especially H_2_O_2_, NO, and H_2_S, within this regulatory framework may further clarify the mechanisms behind fruit ripening control in both climacteric and non-climacteric species.

## Author Contributions

All authors listed have made a substantial, direct and intellectual contribution to the work, and approved it for publication.

### Conflict of Interest Statement

The authors declare that the research was conducted in the absence of any commercial or financial relationships that could be construed as a potential conflict of interest.
